# The Monotonic Behavior of Existing Bridge Piers Retrofitted by Expansive Concrete-Filled Steel Tubes: An Experimental and Numerical Study

**DOI:** 10.3390/ma18225186

**Published:** 2025-11-14

**Authors:** Ganghui Peng, Guowen Yao, Hongyu Jia, Yun Yao, Shixiong Zheng

**Affiliations:** 1 State Key Laboratory of Mountain Bridge and Tunnel Engineering, Chongqing Jiaotong University, Chongqing 400074, China; ganghui.peng@cdutetc.cn; 2 School of Civil Engineering, Chongqing Jiaotong University, Chongqing 400074, China; 3 The Engineering & Technical College of Chengdu University of Technology, Leshan 614000, China; 4 School of Civil Engineering, Southwest Jiaotong University, Chengdu 610031, China

**Keywords:** expansive concrete-filled steel tube, expansive agent, bridge piers, reinforcement technique, experimental and numerical investigations

## Abstract

With the growing traffic volume in China, numerous existing highway bridges in seismic zones are constrained by outdated design standards that are inadequate against current seismic requirements. To address this issue, this study proposes a novel reinforcement technique using expansive concrete-filled steel tube (ECFST) for bridge piers. Through combined experimental and numerical investigations on ECFST columns, the effect of expansive agent (EA) content on steel tube strain was systematically examined. The monotonic quasi-static tests were conducted to evaluate the influence of steel tube thickness, concrete strength, reinforcement thickness, and EA content on the ultimate bearing capacity. The proposed method was implemented in a case study involving a reinforced concrete pier, with analysis focused on the “confinement–self-stress coupling mechanism” of ECFST. Results demonstrated good agreement between numerical simulations and experimental data. The optimal EA content was identified as 15%, achieving the most effective reinforcement. ECFST-reinforced piers exhibited significantly enhanced seismic performance, achieving up to 22.6% increase in peak bearing capacity compared to non-expansive concrete filling. While steel tube thickness considerably affected the reinforcement efficiency, concrete strength grade showed minimal impact. This research provides theoretical support and practical design guidelines for seismic retrofitting of similar bridge piers.

## 1. Introduction

Global transportation infrastructure is increasingly challenged by growing operational demands and natural hazards. The continuous rise in road traffic volume has subjected existing bridges to sustained heavy loads and cumulative fatigue damage [1]. Moreover, many bridges in seismically active regions were designed according to outdated standards, rendering them vulnerable under frequent seismic events [2]. These factors collectively accelerate the performance degradation of existing reinforced concrete (RC) bridge piers, exposing critical seismic deficiencies such as poor ductility, limited energy dissipation capacity, and susceptibility to shear failure [3]. Therefore, developing efficient and reliable strengthening techniques to enhance the seismic performance of existing piers and to ensure the safety and post-earthquake resilience of transportation lifelines has become an urgent priority in civil engineering. The primary objective of this study is to propose a novel strengthening method for bridge piers to enhance their seismic performance.

Researchers worldwide have developed various strengthening methods for bridge piers. Conventional techniques include reinforced concrete jacketing, steel plate bonding, and fiber-reinforced polymer (FRP) wrapping [4]. Li et al. [5] investigated the seismic behavior of textile-reinforced concrete (TRC)-strengthened columns under various influencing factors. Hu [6] conducted axial compression tests on RC short columns strengthened with circular steel tubes and examined their mechanical behavior. Zong et al. [7] applied steel plate bonding to retrofit RC box-section bridge piers. Chen et al. [8] adopted a hybrid strategy using FRP and engineered cementitious composite (ECC) to strengthen earthquake-damaged bridges. Hua et al. [9] used high-performance ferrocement laminate (HPFL) combined with bonded steel plates (BSP) to enhance the performance of RC columns. Benzaamia et al. [10] proposed a deep learning-based approach to predict the compressive strength of circular CFRP-confined concrete columns, considering the influence of column diameter, concrete strength, and FRP thickness. Yehia et al. [11] employed machine learning to analyze parameters affecting the strength, ductility, and failure modes of FRP-strengthened rectangular concrete columns. Narlitepe et al. [12] introduced a new seismic strengthening technique using CFRP combined with structural repair mortar and longitudinal reinforcement. Zhang et al. [13] studied the load-bearing capacity and stiffness of recycled aggregate concrete structural members strengthened with CFRP. Research efforts have employed diverse strategies to improve the performance of concrete-filled steel tubular columns. Wang et al. [14] established that the load-bearing capacity of square sections is significantly enhanced by using steel fiber-reinforced high-strength concrete as the infill material. Alternatively, Deng et al. [15] improved the interfacial bond in UHPC-filled tubes through the strategic use of internally welded steel rings. Ma et al. [16] subsequently developed a predictive model for interfacial bond strength, explicitly incorporating the contributory effects of micro-interlock and friction derived from experimental data. Building upon material innovations, Mohammed et al. [17] verified that a UHPC matrix incorporating both expansive agents and steel fibers offers concurrent improvements in interfacial bond strength and structural load-bearing capacity.

Despite their widespread application, these conventional methods exhibit certain drawbacks. Section enlargement techniques often significantly increase structural self-weight and may cause substantial damage to the original members. Steel plate bonding involves complicated construction processes, and the bond integrity between steel and concrete is prone to deterioration under seismic actions [18,19]. FRP materials behave linearly elastically and often fail to fully activate their confining effect before concrete crushing, leading to brittle debonding or fracture with limited energy dissipation capacity [20,21,22,23]. Despite combining the advantages of both materials—the high strength of FRP and the ductility of ECC—the FRP-ECC hybrid system suffers from several drawbacks, primarily complex construction procedures, high material costs, and uncertainties in long-term performance [24]. The exceptional mechanical properties of UHPC jackets are often counterbalanced by high material costs and complex construction processes, thereby restricting their large-scale application in conventional engineering projects [25]. Additionally, for piers with pre-existing damage, the effectiveness of these methods in restoring and further enhancing seismic resilience remains limited [26].

Cheng et al. [27] examined the expansion and mechanical response under load of short circular steel tubes confined with self-compacting concrete that contained additives. Their findings highlighted the considerable impact of expansion stress on the ultimate load-bearing capacity of these composite columns. In a related study, Shen et al. [28] formulated an expansive ultra-high performance concrete (UHPC) specifically for steel tube confinement. This material achieved a notable circumferential expansion of 308 *με* and generated a confining pressure of 3.65 MPa within a 24 h period. Similarly, BWHA et al. [29] utilized a unique micro-expansive, self-compacting UHPC in stub columns, demonstrating that incorporating expansive agents promotes a robust bond at the steel-concrete interface. Furthermore, Qi et al. [30] applied pre-stress through expansive concrete to actively confine CSFFT composites. Their experimental results showed that this pre-stressing method significantly improves the strength enhancement ratio of the confined specimens.

While previous research has extensively documented the mechanical behavior and excellent composite action of expansive concrete-filled steel tubes (ECFTs), there is scarce documentation regarding their application in strengthening existing bridge piers. In response, this study introduces a novel strengthening technique that utilizes an Expansive Concrete-Filled Steel Tubular (ECFST) jacket. This approach effectively integrates the benefits of traditional concrete-filled steel tube (CFST) systems with the self-stressing characteristics of shrinkage-compensating concrete, thereby bridging a critical gap between research and practical application in pier rehabilitation.

The paper is structured as follows: First, small-scale ECFST specimens are fabricated and tested to determine the optimal EA content, the thickness of the concrete jacket, and the axial load-bearing behavior under various conditions. Second, a finite element (FE) model is developed using ABAQUS (v.6.16), and its predictions are validated against experimental results. A parametric study is then conducted based on the validated model. Finally, a case study of an RC bridge pier strengthened with the ECFST method is carried out using the FE model to verify the effectiveness of the proposed technique.

## 2. Experimental Program

To systematically evaluate the strengthening effectiveness of ECFST on existing damaged bridge piers, this study proposes an integrated methodology combining experimental and numerical analysis. The study aims to investigate the strengthening mechanisms and quantify the performance improvements.

First, material-level tests are conducted. The primary objective is to measure the hoop strain in the steel tube induced by the expansive concrete during hardening, thus directly quantifying the self-stressing effect. By testing mixtures with different expansion agent dosages, the development of hoop strain is analyzed to determine the optimal dosage for the most effective confinement. This provides crucial parameters for subsequent experiments.

Second, component-level tests are performed. Based on the optimal dosage, scaled damaged pier specimens are strengthened with ECFST jackets. To investigate the influence of jacket thickness, multiple scenarios with varying thicknesses are designed. Axial compression tests on both strengthened and unstrengthened control specimens are conducted to precisely measure their ultimate bearing and deformation capacities, thereby comparing the effectiveness of different schemes.

Finally, a numerical model is developed using ABAQUS to simulate material nonlinearity, interface contact, and the expansion effect. The model’s reliability is verified by comparing its predictions (e.g., load–displacement curves, failure modes) with experimental results. The validated model is then used for parametric studies to investigate hard-to-test variables, comprehensively revealing the system’s working mechanism.

We acknowledge that a strict, fully mechanical similarity analysis was not performed when determining the scale ratio. The primary reason is that achieving complete similarity in geometry, material properties, and loading would impose extremely demanding requirements on both model materials and loading equipment, which are difficult to meet under the current experimental conditions. This incomplete similarity can introduce a “size effect,” potentially influencing the results. This influence manifests primarily in the size dependence of concrete properties (such as compressive strength and fracture energy) and scale effects in interface behavior (for instance, the bond-slip at the steel-concrete interface may not scale proportionally). To evaluate these potential influences and demonstrate the reliability of our experimental findings, a combined approach of qualitative verification and quantitative evaluation was employed.

### 2.1. Expansion Strain Experimental

**Figure 1:**
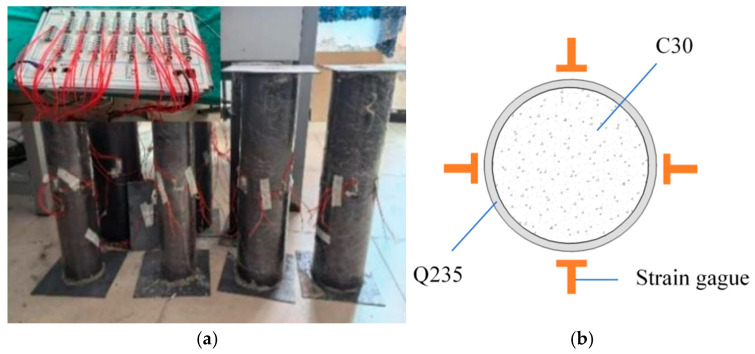
Steel tube strain measurement: (**a**) steel tube-expanded concrete specimen, and (**b**) distribution of strain gauge measurement points.

To investigate the influence of EA dosage in ECFST and to determine its optimal content, we incorporated the EA at ratios of 0%, 5%, 8%, 10%, 15%, and 20%. The dosage of the expansive agent is expressed as a percentage by mass of the cement. The optimal dosage was determined experimentally by measuring the hoop strain of the steel tube. A high-performance EA produced by Sichuan Jiahua Special Cement Factory was used. The concrete was grade C30, with a mix proportion of cement/water/sand/coarse aggregate = 1:0.42:1.11:2.72. The base mix proportion was designed in accordance with the “Specification for Mix Proportion Design of Ordinary Concrete” [31]. The steel tubes were fabricated from Q235 steel, each measuring 500 mm in length, 150 mm in outer diameter, and 2 mm in wall thickness. The expansive concrete-filled steel tube columns were wrapped in sealing membranes. This method created a high-humidity environment (approaching 100% RH) around the specimens by effectively trapping the mix water throughout the 60-day curing period, during which the temperature was maintained at 20 ± 3 °C. As shown in Figure 1, four sets of strain gauges were uniformly mounted around the mid-height section of the specimens to monitor the circumferential strain in the steel tube during concrete expansion.

### 2.2. Axial Compression Test on Columns

**Figure 2:**
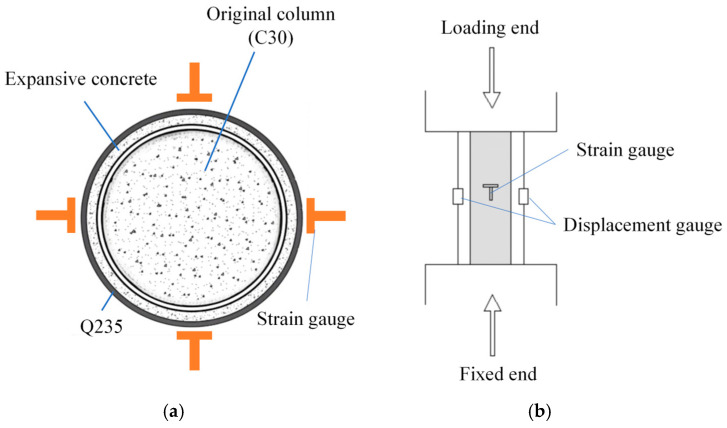
Distribution of strain gauge measurement points on test specimens: (**a**) section, and (**b**) elevation.

The columns were fabricated from the aforementioned C30 concrete, each measuring 20 cm in diameter and 60 cm in height. External confinement was achieved using externally confined steel tubes, with the annular gap between the steel tube and the original column filled with C40 expansive concrete. The experimental variables included three thicknesses of expansive concrete (3 cm, 5 cm, and 7 cm) and three EA dosages (0%, 15%, and 20%); complete details of the parameters are provided in Table 1. As illustrated in Figure 2, four strain gauge rosettes were symmetrically mounted at the mid-height of both the short column and the steel tube to measure the hoop and longitudinal strains in the tube during axial compression. Dial gauges installed diagonally on the outer tube were employed to monitor vertical displacement. To ensure data integrity, strain and displacement readings from identical sensor groups were processed using an equal-interval averaging method.

**Figure 3:**
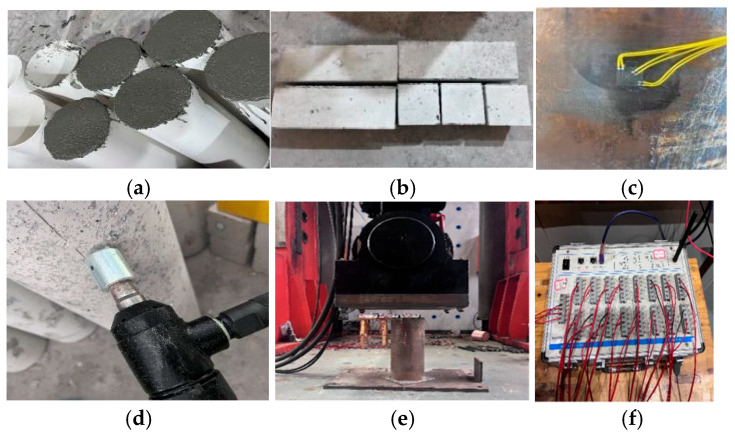
Specimen preparation and loading of ECFST: (**a**) concrete placing; (**b**) concrete test specimens; (**c**) strain gauge bonding; (**d**) surface roughening; (**e**) actuator, and (**f**) data acquisition.

The preparation of the specimens and the loading setup for ECFST are illustrated in Figure 3. The loading procedure employed a dual control strategy based on both force and displacement. Initially, a force-controlled incremental loading method was applied until the specimen reached 70% of the estimated ultimate load, after which the control was switched to displacement-controlled loading. After the peak load was exceeded, loading was terminated under either of the following criteria: (a) the bearing capacity dropped below 80% of the peak load, or (b) macroscopic failure was observed. A stepwise unloading method was subsequently implemented.

Table 2 summarizes the key material properties. At a 20% dosage of EA, the cube compressive strength decreased by 16%, and the axial compressive strength declined by 11%. This reduction in strength is attributed to the development of internal expansion stresses that exceeded the tolerable threshold, leading to extensive microcracking that substantially impaired both the cohesion and load-bearing capacity of the concrete.

## 3. Results and Discussion

This study aims to investigate the short-term mechanical performance and preliminary feasibility of a new ECFST strengthening method, with particular emphasis on identifying the optimal expansive agent content, ultimate load capacity, and failure modes. Although creep and shrinkage critically influence long-term deformation and prestress loss, their impact is considered minimal in the context of the short-term performance evaluated herein [32,33]. Consequently, a comprehensive investigation of long-term performance constitutes the focus of future work.

### 3.1. Expansion Strain

**Figure 4:**
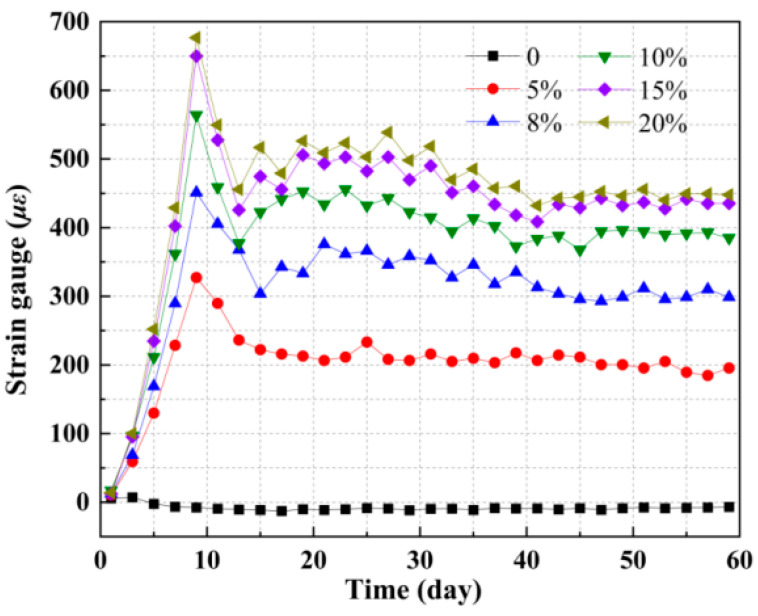
Relationship between hoop strain of steel tube and time.

The concrete free deformation test was conducted in accordance with the “Standard for Test Methods of Long-term Performance and Durability of Ordinary Concrete” (GB/T 50082-2009) [34], using a horizontal concrete shrinkage instrument. The expansion strain evolution in ECFST specimens, monitored for over 60 days (Figure 4), exhibited three distinct nonlinear stages, independent of additive concentration: (i) Initial Stage (Days 1–9): Characterized by rapid strain development. Sufficient internal moisture promoted accelerated cement hydration and reactions of expansive agents, forming ettringite and generating substantial internal swelling stress. This resulted in a swift increase in expansion strain that significantly outweighed any autogenous shrinkage. The exothermic nature of hydration further contributed through induced thermal expansion [35,36]. (ii) Intermediate Stage (Days 9–38): Marked by a decelerating strain rate. As free water content diminished, hydration slowed, reducing the driving force for expansion. Concurrently, the developing micropore structure provided increasing restraint against swelling. Capillary water loss generated negative pressure, causing shrinkage that partially offset the ongoing expansion. These processes, sensitive to ambient conditions, led to noticeable strain fluctuations [37,38]. (iii) Late Stage (After Day 38): Defined by strain stabilization. Following the near-completion of hydration and exhaustion of expansive agents, an equilibrium was reached between the residual swelling stress and the microstructural resistance. The system became stable, with micro-deformation becoming negligible and insensitive to environmental variations [39].

In the absence of EA (0% by mass), both strain and induced self-stress displayed negative values, consistent with typical concrete shrinkage. Expansion strain increased notably when the EA content was below 10%. However, as the EA content exceeded 15%, the rate of expansion strain growth decreased significantly, yielding only a marginal expansion effect. Specifically, increasing the EA content from 15% to 20% enhanced the expansion strain by merely 3%. This limited enhancement can be attributed to two main factors: firstly, the reactivity gain associated with the finite microstructural activity gradually weakened, and the active components became depleted, resulting in insufficient reactants for further expansion and thus restricting additional strain development. Secondly, at 20% EA, pre-crystallization caused microstructural densification, which inhibited radial expansion and promoted internal compression. This contrasts with the behavior at 15% EA, where the key mechanism was the occupation of critical pore spaces by expansion products. In summary, it can be determined that the optimal dosage of this EA for ECFST is 15%.

### 3.2. Load–Displacement Curve

**Figure 5:**
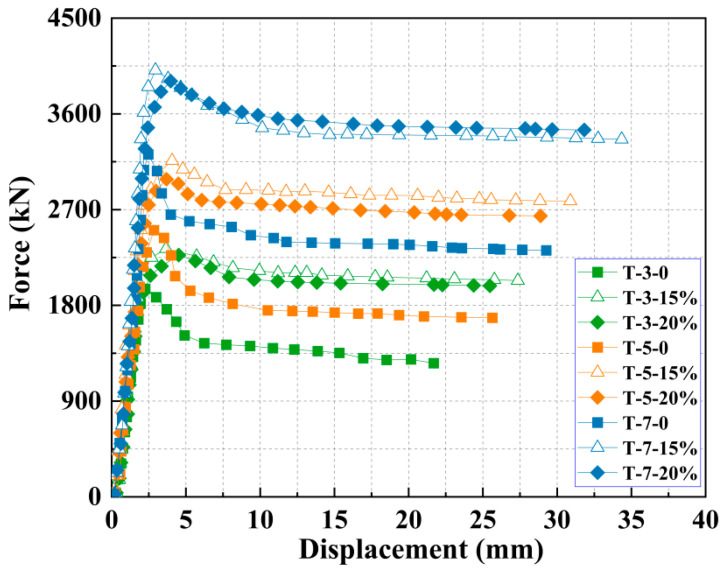
Comparison of load–displacement curves of piers with varied retrofitting schemes.

The load–displacement curve was obtained by applying quasi-static loading to small-scale specimens. Figure 5 presents the load–displacement behavior of ECFST columns with different mix designs. Table 3 presents the ultimate displacement and peak load for different structural members. In the initial loading phase, all curves show nearly linear elastic response. After yielding, the behavior transitions to elastic–plastic. At the same concrete thickness, the load–displacement curves with different EA contents are almost identical within the 0–1 mm range, maintaining linear proportionality, indicating minimal prestress effect during this matrix-controlled phase.

Notable differences appear at 1–2 mm displacement: the 0% EA curve rises progressively slower with a reducing slope, while both 15% and 20% EA curves maintain steeper slopes with faster load increase. This demonstrates that EA helps preserve specimen stiffness during this phase, delaying plasticity onset. The mechanism lies in the fact that the self-induced stress from expansion enhances the composite action between the concrete core and the steel tube. The EA-containing curves show clearly higher stiffness and peak loads versus the EA-free case. The curve for 0% EA peaks at about 2 mm and then drops sharply, while the EA curves peak at 3–4 mm and exhibit a more extended, gradual post-peak descent. This confirms that EA improves steel–concrete compatibility, enhancing overall stiffness, ductility, and ultimate capacity. Critically, the 15% EA mixture exhibits greater stiffness and a higher peak load than the 20% mixture, indicating that the effectiveness of the EA does not increase monotonically with its dosage. An overabundance of EA induces internal stresses surpassing the concrete’s tensile strength, thereby initiating premature microcracking. This micro-damage progressively compromises the material’s compressive resistance and leads to a net reduction in reinforcement efficacy.

Under a fixed EA content but varying thicknesses of the expansive concrete reinforcement, the three curves nearly coincide during the initial loading stage (0–1 mm). It indicates that at trim displacement levels, the stiffness of specimens with different reinforcement thicknesses is comparable, and all remain in the elastic stage, where the load and displacement exhibit an approximately linear relationship, and the material deformation is predominantly elastic. As displacement increases, the three curves begin to diverge. The curve for the 3 cm reinforcement thickness shows a slower rate of load increase, reflecting a reduction in stiffness and a transition into the elasto-plastic stage. In contrast, the curves for the 5 cm and 7 cm reinforcement thicknesses maintain relatively higher slopes, delaying the entry into the plastic stage. This is attributed to the larger load-bearing area provided by the greater reinforcement thickness, which allows the reinforcement layer to carry a greater share of the load and imposes a stronger confinement effect on the original concrete column, thereby more effectively enhancing the overall structural performance. With increasing reinforcement thickness, the peak load of each curve rises sequentially, and the displacement corresponding to the peak load also increases progressively. It demonstrates that increasing the reinforcement thickness not only improves the load-bearing capacity of the specimen but also enhances its ductility, enabling the structure to undergo greater deformation and absorb more energy before failure.

### 3.3. Load-Strain Curve

**Figure 6:**
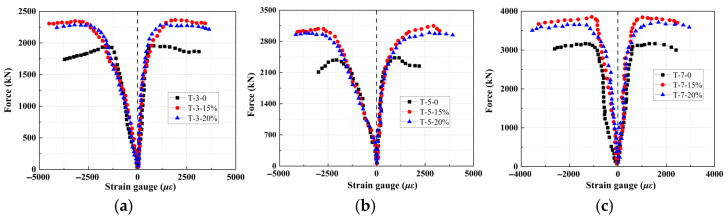
Load–strain curves under axial compression with different EA dosage: (**a**) 3 cm, (**b**) 5 cm, and (**c**) 7 cm.

To ensure measurement accuracy, four circumferential and axial strain gauges were uniformly distributed around the steel tube to monitor strain development during loading, as shown in Figure 2. The average values were used as the final results, with negative and positive values denoting axial and circumferential strain, respectively. Figure 6 and Table 4 reveal the following observations: (1) During the initial loading stage, the hoop strain remains close to zero, whereas the axial strain is significantly higher. At this point, the longitudinal bond friction between the steel tube and concrete governs the strain behavior, while the confining effect of the steel tube is not yet pronounced. (2) As loading progresses into the elasto-plastic stage, both strain components exhibit nonlinear growth, with the hoop strain increasing at a notably higher rate. This stage is characterized by the development of a triaxial stress state in the concrete core due to significant lateral expansion, during which the steel tube’s confining effect becomes active, effectively restraining concrete dilation and enhancing its compressive strength. (3) For a fixed EA content, increasing the thickness of the expansive concrete reinforcement prolongs the elastic stage. Differences in axial strain emerge early in the loading process, while hoop strains remain similar across specimens during the elastic stage. Divergence in hoop strain only becomes evident upon entering the elasto-plastic stage. (4) Under the same reinforcement thickness, specimens with 15% and 20% EA exhibit a longer elastic stage compared to the group without EA. Furthermore, the specimen with 15% EA achieves a higher ultimate axial load capacity than that with 20% EA. This confirms that 15% represents the optimal EA content for the expansive concrete, and again verifies that excessive EA can damage the internal microstructure of the concrete, thereby reducing its load-bearing capacity.

### 3.4. Model Validation

**Figure 7:**
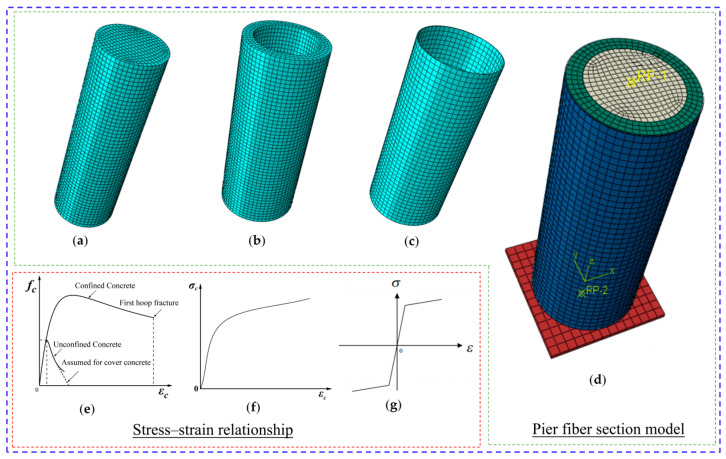
Finite element model of the specimens: (**a**) original column, (**b**) expansive concrete, (**c**) steel tube, (**d**) assembly, (**e**) ordinary concrete, (**f**) expansive concrete and (**g**) steel tube.

A numerical finite element model for short columns composed of steel tubes and expanding concrete reinforcement was developed utilizing ABAQUS software [40]. The concrete and loading plate were simulated using C3D8R solid elements, while the steel tube was simulated using S4R shell elements, with a validated mesh size of 8 mm. The constitutive model for concrete was based on the Mander model, and the Kent-Scott-Park model was employed for steel. The steel material incorporates kinematic hardening to accurately capture the Bauschinger effect. The expansion effect of expansive concrete was simulated by applying thermal expansion strain. In the model, surface-to-surface contact was defined between the steel tube and the concrete, while interface debonding was not considered. A tie constraint was adopted at the interface between the expansive concrete and the ordinary concrete, ensuring the two surfaces were perfectly bonded, thereby accurately representing their interaction. The architecture of the finite element modeling is illustrated in Figure 7.

#### 3.4.1. Failure Mode Comparison

**Figure 8:**
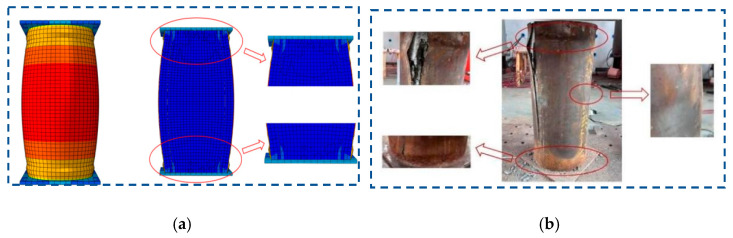
Failure modes of short columns: (**a**) numerical model, and (**b**) experimental.

The simulated failure modes illustrated in Figure 8 show close agreement with the experimental results under axial compression. Structural failure is characterized by buckling of the upper and lower steel tubes—which detached from the inner concrete—lateral bulging in the mid-height region, and crushing of the upper concrete due to compressive overload. Cracks observed during testing originated from inconsistencies in weld strength within the fabricated tubes, resulting in premature failure at localized weak points under stress.

**Figure 9:**
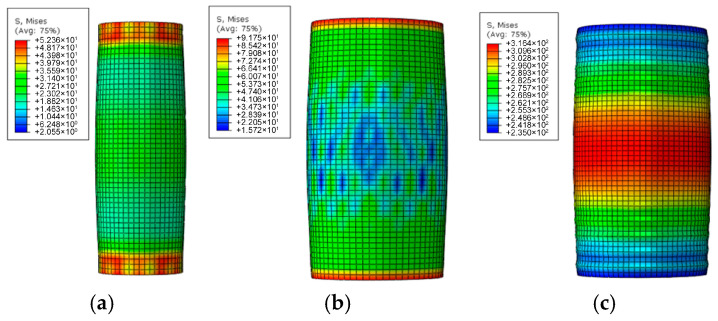
Stress distribution in piers during plastic phase: (**a**) concrete column, (**b**) retrofitting layer, and (**c**) steel tube.

Figure 9 illustrates the stress distribution in the plastic stage. It can be observed that the stress in the expansive concrete significantly exceeds that in the core concrete, validating its role in enhancing the load-bearing capacity through confinement. Meanwhile, the steel tube yields at a stress of 316.4 MPa, fully mobilizing its plastic deformation capacity. The high stress level in the steel tube indicates effective restraint of the circumferential deformation of the expansive concrete, thereby demonstrating the effectiveness of the synergistic interaction between the expansive concrete and the steel tube in strengthening the column.

#### 3.4.2. Load–Displacement Curves Comparison

**Figure 10:**
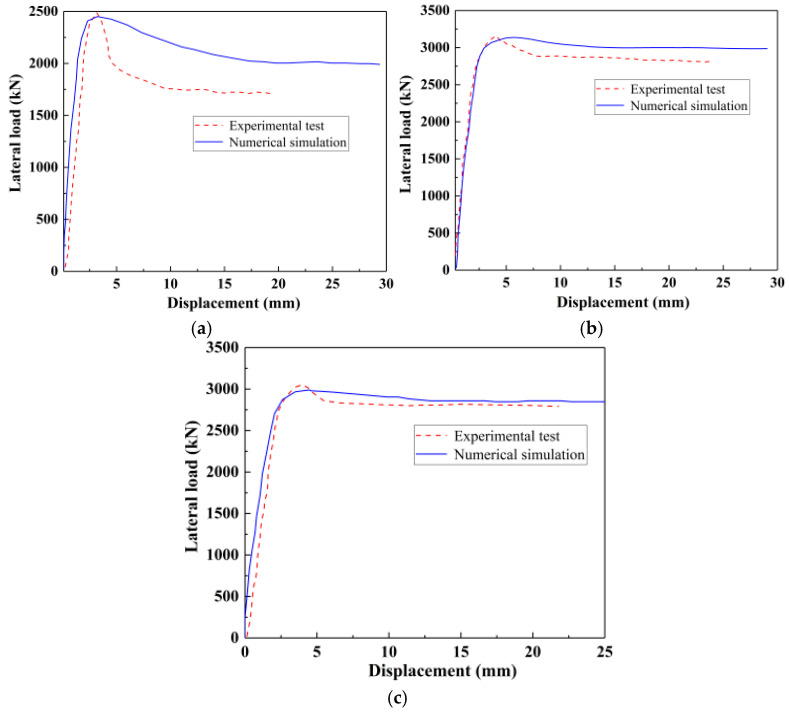
Comparison of load–displacement curves: (**a**) T-5-0, (**b**) T-5-15%, and (**c**) T-5-20%.

Finite element simulation of the ECFST-reinforced column in ABAQUS demonstrated strong correlation with experimental data, as illustrated in Figure 10 and Table 5. The numerical model accurately reproduced both the overall trend and peak load capacity of the physical tests (*R*^2^ = 0.9964), indicating minimal systematic error in the modeling approach.

Notable differences included a moderately reduced initial stiffness and enhanced post-peak load retention in the simulated response, contrasting with the sharper degradation observed experimentally following ultimate displacement. These variations primarily stem from inherent material heterogeneities and boundary condition effects present in physical testing conditions. Conversely, the finite element model incorporates idealized material representations that exclude natural defects and localized damage mechanisms, resulting in a more gradual post-yield softening response and moderately elevated residual strength predictions.

#### 3.4.3. Analysis of Ultimate Load-Bearing Capacity

The calculation of the axial compressive bearing capacity of concrete-filled steel tubes is usually based on the ultimate theory [41,42]. The strength enhancement coefficient *k* is introduced to account for the axial compressive bearing capacity of expansive concrete-filled steel tube columns, considering the influence of self-stress [43,44]. The theoretical calculation results are compared with the experimental data, as shown in Table 3. It shows that the ultimate bearing capacities predicted by ultimate theory are in close agreement with the experimental results, with deviations within 10%. The inherent heterogeneity of concrete, combined with residual stresses and micro-structural defects in steel, causes actual engineering materials to deviate significantly from idealized models, resulting in a discrepancy between theoretical predictions and actual structural load-bearing capacity.

Table 5 also indicates that the use of 15% and 20% EA improved the ultimate bearing capacity of the specimens in a nonlinear manner. Specimens T-3-15, T-5-15, and T-7-15 exhibited ultimate bearing capacities that were 18.8%, 27.7%, and 24.1% higher than those of the control groups T-3-0, T-5-0, and T-7-0, respectively. Similarly, specimens T-3-20, T-5-20, and T-7-20 surpassed the controls by 16.7%, 22.5%, and 16.8%, respectively. The results indicate that using a steel tube with a 5 cm wall thickness provides the greatest improvement in bearing capacity. Moreover, the incorporation of 15% EA yields slightly better enhancement compared to 20%. Excessive EA content (>15%) induces abnormal tensile strains, promoting detrimental microcracks that compromise concrete integrity and reduce compressive strength. Therefore, the optimal retrofit strategy combines 15% EA with an expansive concrete thickness of 5 cm. This provides an experimental basis for the reinforcement of bridge piers using steel tube-expanded concrete. The experimental investigation here focuses on small-scale components.

Experimental results confirm a superior strength-ductility synergy in the ECFST system, which exhibits a 104.6% increase in ultimate load capacity—the highest among the methods compared. This enhancement is attributed to the active confinement from the expansive concrete, a mechanism that fully mobilizes the steel tube’s tensile capacity and induces a beneficial triaxial stress state in the core concrete. In contrast, FRP wrapping significantly improves strength but offers limited ductility gains. While UHPC jacketing provides high stiffness and strength, it does so at a substantially higher material cost. Conversely, ECC exhibits exceptional deformation capacity but only moderate strength enhancement. Therefore, the ECFST system presents a more balanced and robust solution for seismic retrofitting, effectively bridging the gap between strength-oriented and ductility-focused strategies.

### 3.5. Parameter Analysis

Piers were retrofitted using ECFST, a method whose mechanical performance is influenced by multiple factors. Based on the finite element model validated in Section 3.4, a parametric analysis was conducted on ECFST-strengthened piers with an EA content of 15% and a uniform strengthening layer thickness of 5 cm. The load–displacement curve was obtained using monotonic quasi-static analysis. The investigated parameters included the wall thickness of the steel tube, along with the strength grades of both the expansive concrete and the steel. The parametric design is summarized in Table 6.

#### 3.5.1. Thickness of the Steel Tube

A parametric analysis was conducted to evaluate how the wall thickness of the steel tube influences the mechanical performance of the retrofitted members, given that thicker tubes provide greater lateral confining pressure. In this study, the wall thickness was varied sequentially at 2, 3, 4, and 5 mm. The following material properties were kept constant across all models: the core concrete was grade C30, the expansive concrete strengthening layer was grade C40 with a 15% EA content, and the steel tube was grade Q235.

**Figure 11:**
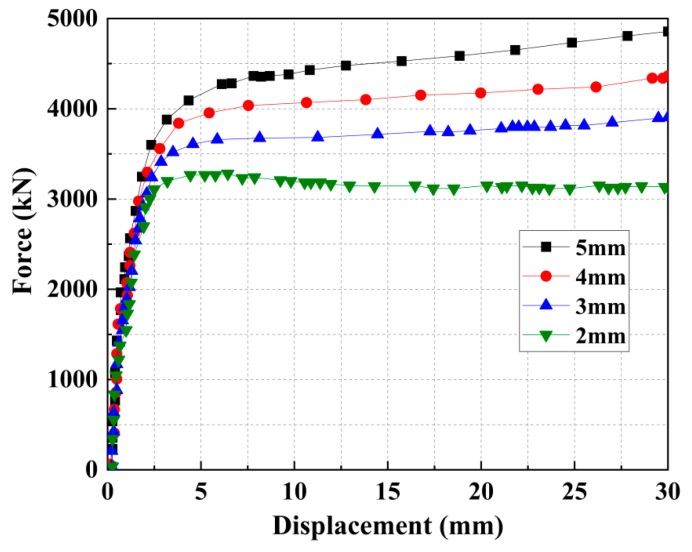
Force–displacement curves of columns under different steel tube thicknesses.

As shown in Figure 11 and Table 7, increasing the steel tube wall thickness significantly enhanced the load-bearing capacity of the ECFST-strengthened columns, with each 1 mm increase contributing to an average gain of approximately 300 kN. In specimens with 3, 4, and 5 mm thick tubes, the load–displacement curves displayed no descending branch but a continuous load increase, demonstrating sustained load resistance under larger deformations. This behavior reflects markedly improved ductility, resulting from the more effective confinement of the concrete core by the thicker steel tube.

#### 3.5.2. Strength of Steel

A series of models were established with the steel yield strength as the variable, assigned the grades Q235, Q345, Q390, and Q420. Across all models, the steel tube thickness was 2 mm, the core concrete was grade C30, and the strengthening layer was C40 expansive concrete with 15% EA.

**Figure 12:**
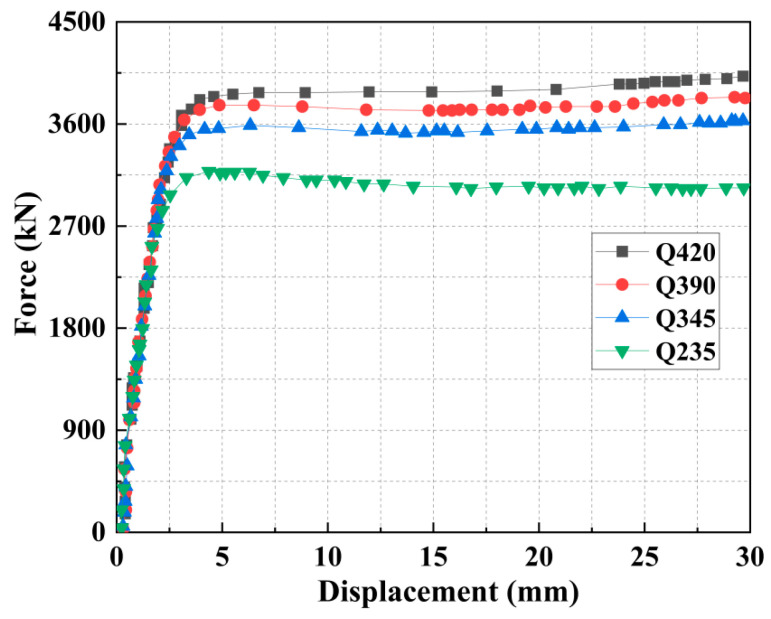
Force–displacement curves of columns under different steel strengths.

As shown in Figure 12 and Table 7, the ultimate load capacity of the specimens increases with the steel grade, a trend attributed to the associated higher yield strength. This provides enhanced hoop confinement during concrete expansion, placing the core concrete in a more pronounced triaxial stress state and thereby improving its compressive capacity. In contrast, the initial stiffness remains largely unaffected by the steel grade, ruling out its practical use for stiffness enhancement. Furthermore, enhancing the steel strength provides only a limited and marginal improvement in the ductility of the pier.

#### 3.5.3. Strength of Concrete

This set of models investigated the influence of the expansive concrete strength grade on structural mechanical performance, with the grade of the strengthening layer being varied as C30, C40, C50, and C60. The following parameters were held constant: core concrete strength of C30, steel tube grade of Q235, and an EA content of 15% in the strengthening layer.

**Figure 13:**
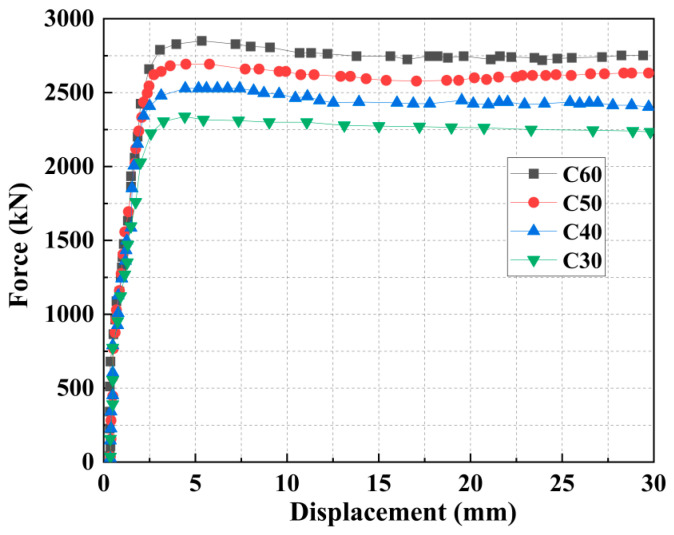
Force–displacement curves of columns under different concrete strengths.

As shown in Figure 13 and Table 7, increasing the concrete strength grade improves the member’s load capacity and stiffness, albeit with diminishing returns on capacity at higher grades. Each 10 MPa increase raised the load capacity by approximately 12%, 6.1%, and 5.5%, with corresponding stiffness gains of 4.2%, 4.7%, and 5.7%. In contrast, the C50 and C60 grades exhibit a distinctive post-peak response characterized by an initial drop followed by a slight recovery. This behavior results from the initial brittleness of high-strength concrete, with the subsequent recovery driven by synergistic interaction between expansion pressure and steel tube confinement.

### 3.6. Case Study

**Figure 14:**
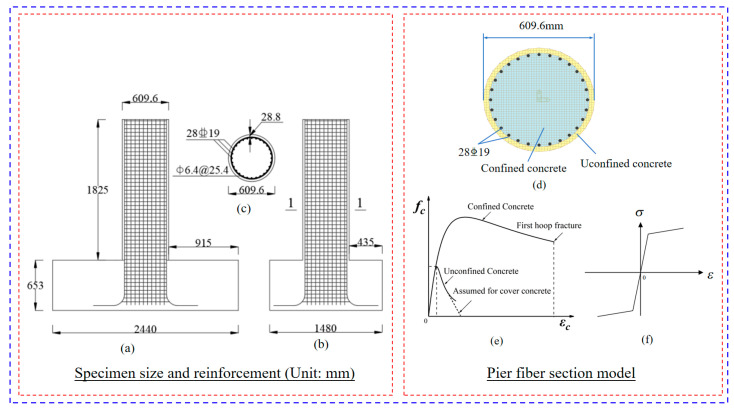
Specimen size and reinforcement: (**a**) front view, (**b**) side view, (**c**) 1-1 cross section, (**d**) pier section, (**e**) concrete fiber and (**f**) rebar fiber.

**Figure 15:**
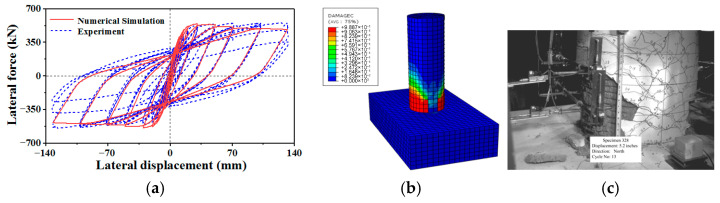
Hysteretic response of the piers under lateral loading: (**a**) hysteretic curve; (**b**) FE model and (**c**) experimental results.

The piers of the case-study bridge are composed of C35 grade concrete. To facilitate comparative analysis and leverage existing experimental benchmarks, the experimental data for piers reported by Calderone et al. [45] were adopted. The pier dimensions, reinforcement details, fiber element modeling of the piers, as well as the constitutive relationships of concrete and steel reinforcement, are all presented in Figure 14. The mechanical properties of the concrete and steel reinforcement are listed in Table 8. A finite element model was developed in ABAQUS, wherein the concrete was modeled with C3D8R elements and the reinforcing steel with T3D2 elements. The reinforcement was embedded within the concrete mesh using the “Embedded” technique. The force–displacement curve was generated by applying a lateral load to the top of the pier following a cyclic loading protocol. As shown in Figure 15, the numerical simulation results exhibit a high degree of agreement with the experimental curve. The constitutive model, validated through this process, demonstrates high reliability and is therefore suitable for subsequent simulation of steel tube-expanded concrete strengthening for the piers.

The strengthening thickness was designed based on its ratio to the pier radius. Following the scaling principle established in Section 2 (from a 10 cm radius model with thicknesses of 3, 5, and 7 cm, giving ratios of 0.3, 0.5, and 0.7), the case study pier with a radius of 30.48 cm was strengthened with expansive concrete-filled steel tubes of 9, 15, and 21 cm in thickness. Based on the content presented in Section 3.1, Section 3.2, Section 3.3, Section 3.4 and Section 3.5, the expansive concrete was designed with a strength grade of C40. The dosage of the EA was set at 0% and 15%, respectively, and the expansion effect was simulated using a temperature increase effect. The steel tube was fabricated from Q235 grade steel, having a wall thickness of 3 mm.

**Figure 16:**
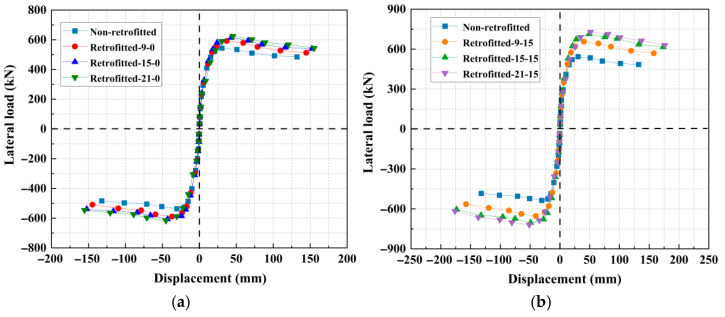
Load–displacement curves of the piers with varying strengthening thicknesses and EA contents: (**a**) an EA content of 0%, and (**b**) an EA content of 15%.

The load–displacement curves, which reflect the ultimate bearing capacity and deformation capacity of the specimens, are presented in Figure 16 for the different strengthening schemes. The label “retrofitted-9-15” denotes a retrofitted concrete thickness of 9 cm and an EA content of 15%, respectively. All curves exhibit a similar trend: an initial linear-elastic response, followed by a plateau as the load approaches its peak, and a subsequent descending branch. The unstrengthened pier (control specimen) demonstrated the poorest performance, characterized by the lowest peak load and initial stiffness. Its load capacity dropped abruptly after a displacement of approximately 30 mm, accompanied by significant residual deformation, which indicates severe stiffness degradation and a rapid loss of bearing capacity following concrete cracking. The application of ECFST significantly improved the structural integrity through the confining effect of the steel tube. At thicknesses of 9 cm, 15 cm, and 21 cm, the peak load increased by approximately 8.84%, 13.14%, and 14.74%, respectively, relative to the control specimen. The incorporation of a 15% EA enhanced the peak load by 20.81%, 31.24%, and 34.04% over the control for the respective thicknesses. Furthermore, compared to the non-expansive, the EA provided additional peak load gains of 11.97%, 18.34%, and 14.96%, alongside improvements in ultimate displacement of 9.86%, 16.14%, and 15.82%. These enhancements are attributed to the self-stress induced by volumetric expansion, which improved the steel-concrete interfacial bond, minimized slip, and resulted in superior composite action. In conclusion, strengthening with ECFST is a highly effective method for enhancing both the bearing capacity and ultimate displacement of existing piers. Nevertheless, in practical engineering, when the thickness of the expansive concrete strengthening layer exceeds a certain threshold, the improvement in the pier’s ultimate load-bearing capacity becomes marginal. This is because an excessively thick strengthening layer significantly increases the localized stiffness, creating a substantial stiffness disparity with the unstrengthened section. Under seismic action, this imbalance can cause the failure location to shift upward from the pier base to a weaker zone with relatively lower stiffness, and cause damage at that point.

### 3.7. Analysis of the Strengthening Mechanism

The significant improvement in strength and displacment is primarily attributable to the effective confinement of the expansive core concrete by the steel tube. Under axial compression, the concrete’s lateral dilation actively presses against the tube, generating confining pressure that places the concrete in a triaxial stress state, thereby suppressing transverse tensile strains and delaying crushing. The development of a triaxial stress state within the confined concrete is the key mechanism behind its improved performance. According to classical concrete mechanics (e.g., as described by the Kent-Park or Mander’s model), concrete under triaxial compression exhibits markedly higher compressive strength and ultimate strain than under uniaxial stress. The expansive nature of the concrete promotes a more uniform and earlier development of this confining pressure. This leads to a more efficient utilization of both the concrete’s compressive capacity and the steel tube’s tensile capacity. The compatibility between the steel tube and the expansive concrete is crucial for composite action. The initial chemical expansion creates a ‘pre-stress’ effect at the interface, ensuring a tight bond upon loading. This effect mitigates the initial gap formation typical of conventional CFSTs, resulting in a stiffer initial response and more immediate confinement development under axial load. Furthermore, the sustained compatibility under increasing load prevents premature local buckling of the steel tube, allowing it to achieve its full tensile yield strength and maintain confinement through large deformations.

**Figure 17:**
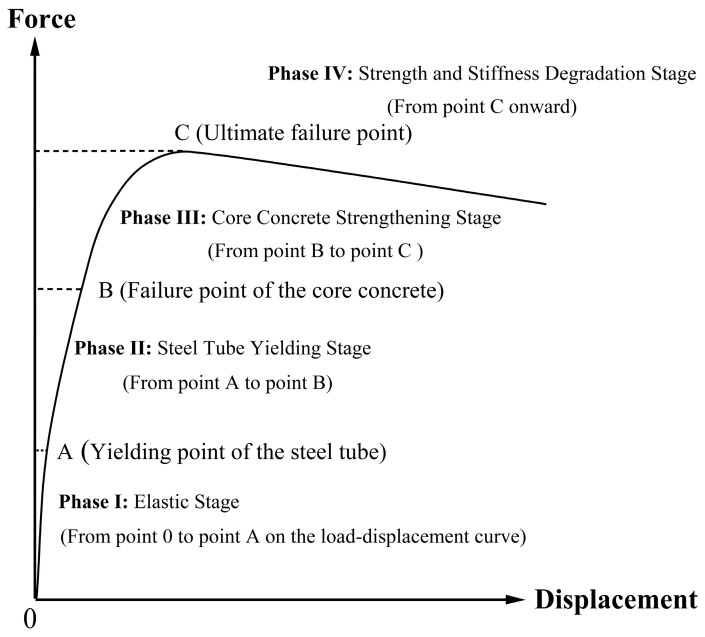
Schematic diagram of the Quasi-Static testing phases for a bridge pier retrofitted with ECFST.

Analysis of the monotonic quasi-static testing and simulations on ECFST-retrofitted bridge piers identifies five characteristic behavioral phases (Figure 17): Phase I, the Elastic Stage, features collaborative work between the retrofit layer and the original pier with fully recoverable deformation; Phase II, the Steel Tube Yielding Stage, begins as the outer steel tube yields and micro-cracks initiate, while the core concrete remains confined; Phase III, the Core Concrete Strengthening Stage, is marked by pronounced confining action, inducing further strength gain and substantial energy dissipation despite concrete plasticity; Phase IV, the Strength Degradation Stage, involves local steel tube buckling/tearing and concrete crushing, leading to a post-peak descent in load capacity; and Phase V, the Ultimate Failure Stage, culminates in structural instability as the load capacity drops to 85% of its peak value.

In summary, through optimized design utilizing the optimal EA content and reinforcement thickness, the column exhibits a significant enhancement in both load-bearing capacity and displacement ductility. The underlying mechanisms can be attributed to the following aspects: (1) Dual Confinement Effect: The external steel tube provides effective passive confinement to the core concrete, substantially improving its compressive strength and deformability. (2) Self-Stress Effect: During hardening, the expansive concrete filler undergoes radial expansion, which induces beneficial pre-compressive stress in the external steel tube and simultaneously establishes favorable self-compressive stress within the concrete. Such an interaction places the reinforcement system under prestress from the initial loading stages. As a result, the confinement effect is activated earlier and more efficiently, which in turn delays local buckling of the steel tube and cracking of the concrete. (3) Synergistic Working Mechanism: The composite system integrates the high ductility and energy dissipation capacity of the steel tube with the high compactness of the expansive concrete, leading to a coordinated improvement in strength, stiffness, ductility, and energy dissipation capacity of the reinforced pier. This results in excellent damage control capability and post-earthquake recoverability.

## 4. Conclusions

This study proposes a novel scheme for strengthening bridge piers using the ECFST technique. The mechanical properties of materials and structural members were systematically investigated through an integrated approach combining experimental testing and numerical simulation. Taking a real-world engineering project as a background, a case study pier was retrofitted with the ECFST system and subjected to quasi-static numerical simulation analysis. The principal conclusions are as follows:

(1) Expansive Agent (EA) Content: The expansive agent significantly improves structural stiffness, load-bearing capacity, and ductility. An optimal EA content of 15% was established, beyond which further additions provide diminishing returns in expansion strain while adversely affecting concrete microstructure and overall member performance. Increasing the thickness of the expansive concrete layer significantly improves stiffness and load-bearing capacity.
(2) Theoretical Validation: The ultimate capacity calculation method based on limit theory demonstrates strong agreement with experimental results, confirming its reliability for evaluating similar ECFST-strengthened columns.
(3) Parametric Influence: The load-bearing capacity increases monotonically with steel tube thickness, as thicker tubes provide enhanced confinement. In contrast, high-strength steel offers only limited improvements to initial stiffness and ductility, despite increasing load capacity. Similarly, gains in structural performance from higher concrete strength in the retrofit layer diminish beyond a certain threshold.

Building on this research, future work should address the following areas:

Long-Term Performance and Durability: Examine behavior under sustained loading and environmental exposure, including long-term creep, shrinkage, and steel tube corrosion effects. Dynamic and Seismic Response: Extend analysis to dynamic and low-cycle fatigue loading to fully evaluate seismic performance and energy dissipation capacities. Advanced Numerical Simulation: Develop refined finite element models that accurately capture interface behavior between the original pier and ECFST layer, along with concrete damage progression under high stress. Sustainability and Economic Viability: Perform comprehensive life-cycle assessments and environmental impact evaluations to optimize the scheme for large-scale implementation.

The ECFST system is an ideal solution for large-scale retrofitting, offering exceptional mechanical properties synergized with superior constructability, cost-effectiveness, and sustainability. Its implementation is underpinned by established CFST and steel jacketing techniques, enabling rapid deployment with minimal site disruption. The method provides pronounced cost benefits through optimized material use, reduced construction time, and minimal need for heavy machinery or formwork. These factors collectively lower labor, equipment, and long-term maintenance expenses. Furthermore, ECFST enhances sustainability by lowering embodied carbon through efficient material utilization and ensuring resource conservation via its extended service life. Its applicability across complex sites without scarce materials makes it a versatile, scalable strategy for regional seismic resilience upgrades.

## Figures and Tables

**Table 1:** Key parameters of retrofitted test specimens.

Specimen ID	Original Column Diameter (cm)	Expansive Concrete Thickness (cm)	Steel Tube Wall Thickness (mm)	EA (%)
T-0-0	20	0	0	0
T-3-0	20	3	2	0
T-3-15	20	3	2	15%
T-3-20	20	3	2	20%
T-5-0	20	5	2	0
T-5-15	20	5	2	15%
T-5-20	20	5	2	20%
T-7-0	20	7	2	0
T-7-15	20	7	2	15%
T-7-20	20	7	2	20%

**Table 2:** Mechanical properties of materials.

Material	Cube Compressive Strength (Mpa)	Axial Compressive Strength (Mpa)	Yield Strength (Mpa)	Ultimate Strength (Mpa)	Elastic Modulus (Gpa)
C30	36.4	24.8	-	-	30.9
C40-0	49.8	32.4	-	-	34.5
C40-15%	50.8	34.3	-	-	34.7
C40-20%	42.9	29.1	-	-	33.2
Q235	-	-	257.7	370.2	198

**Table 3:** Ultimate displacement and peak load of different specimens.

Specimen ID	Ultimate Displacement (mm)	Peal Load (kN)
T-3-0	21.68	1955
T-3-15	27.35	2332
T-3-20	25.47	2273
T-5-0	25.64	2507
T-5-15	30.89	3161
T-5-20	28.89	2986
T-7-0	29.27	3218
T-7-15	34.35	4011
T-7-20	31.83	3907

**Table 4:** Peak load and strain of different members under axial compression.

Specimen ID	Peal Load (kN)	Axial Strain (με)	Circumferential Strain (με)
T-3-0	1957	3720	3126
T-3-15	2363	4500	3441
T-3-20	2298	4111	3642
T-5-0	2431	2997	2198
T-5-15	3077	4080	3249
T-5-20	2992	4164	3924
T-7-0	3166	2618	2408
T-7-15	3843	3280	2459
T-7-20	3670	3544	2955

**Table 5:** Peak load of the columns under different strengthening strategies.

Specimen ID	Experimental Value *N_m_* (kN)	Simulated Value *N_s_* (kN)	Ultimate Theory Value *N_j_* (kN)	Relative Error (%)	
T-3-0	1960.35	1937.76	1963.920	1.152	0.182
T-3-15	2329.83	2336.28	2544.458	0.277	9.212
T-3-20	2287.21	2262.17	2480.521	1.095	8.452
T-5-0	2465.12	2450.66	2497.012	0.587	1.294
T-5-15	3149.12	3130.08	3275.630	0.605	4.017
T-5-20	3019.31	2988.96	3189.879	1.005	5.649
T-7-0	3232.13	3162.44	3091.132	2.156	4.362
T-7-15	4011.09	4041.93	4096.853	0.769	2.138
T-7-20	3776.21	3884.19	3986.090	2.859	5.558

**Table 6:** Design parameters of the pier.

Parameters	Components	D × h (mm × mm)	t (mm)	$f_{y}$ (Mpa)	$f_{\mathrm{cu}}$ (Mpa)
Thickness of the steel tube	M1-2-Q235-C40	300 × 600	2	235	40
	M2-3-Q235-C40	300 × 600	3	235	40
	M3-4-Q235-C40	300 × 600	4	235	40
	M4-5-Q235-C40	300 × 600	5	235	40
Strength of steel	M5-2-Q235-C40	300 × 600	2	235	40
	M6-2-Q345-C40	300 × 600	2	345	40
	M7-2-Q390-C40	300 × 600	2	390	40
	M8-2-Q420-C40	300 × 600	2	420	40
Strength of concrete	M9-2-Q235-C30	300 × 600	2	235	30
	M10-2-Q235-C40	300 × 600	2	235	40
	M11-2-Q235-C50	300 × 600	2	235	50
	M12-2-Q235-C60	300 × 600	2	235	60

Note: D and h represent the inner diameter and height of the steel tube, respectively; *t* is the thickness of the steel tube. Taking the designation M1-2-Q235-C40 as an example: “M1” denotes model number 1, “2” indicates a steel tube thickness of 2 mm, “Q235” signifies a steel yield strength of 235 MPa, and “C40” represents an expansive concrete compressive strength of 40 MPa.

**Table 7:** The bearing capacity and initial stiffness of components.

Parameters	Components	Bearing Capacity (kN)	Initial Stiffness (mm)
Thickness of the Steel Tube	M1-2-Q235-C40	3191.08	3314.23
	M2-3-Q235-C40	3545.66	3646.41
	M3-4-Q235-C40	3862.71	3964.09
	M4-5-Q235-C40	4096.95	4305.53
Strength of Steel	M5-2-Q235-C40	3191.08	3324.18
	M6-2-Q345-C40	3599.17	3322.92
	M7-2-Q390-C40	3773.44	3314.16
	M8-2-Q420-C40	3867.13	3311.44
Strength of Concrete	M9-2-Q235-C30	2850.89	3195.83
	M10-2-Q235-C40	3191.08	3327.28
	M11-2-Q235-C50	3386.19	3483.49
	M12-2-Q235-C60	3573.81	3685.62

**Table 8:** Physical and mechanical properties of material.

Strength (Mpa)			Modulus of Elasticity (Mpa)	
*f_y_*	*f_yv_*	*f_cu_*	*E*_1_	*E*_2_
441.3	441.3	34.5	3.15 × 10^4^	2.0 × 10^5^

## Data Availability

The original contributions presented in this study are included in the article. Further inquiries can be directed to the corresponding author.

## References

[1] Jia H.Y., Xiao C.Z., Kang W., Wang C.Q., Zheng S.X. (2025). Review of research on vulnerability of transportation infrastructure to extreme climatic conditions. J. Southwest Jiaotong Univ..

[2] Wu J., Wu H.X., Yang Y., Wen D.C. (2025). Investigation and mechanism analysis of bridge damage in the 2021 Maduo earthquake, Qinghai province. World Earthq. Eng..

[3] Hu Z.L., Wei B., Jiang L.Z., Li S.S. (2023). Research on damage quantification of high-speed railway bridge piers. China Civ. Eng. J..

[4] China Forum for UN Procurement (2024). Technical Specification for Strengthening Engineering of Urban Bridges.

[5] Li Y., Yin S., Dai J., Liu M. (2020). Numerical investigation on the influences of different factors on the seismic performance of TRC-strengthened RC columns. J. Build. Eng..

[6] Hu X. (2015). Research on Compressive Performance and Bearing Capacity of RC Short Columns Strengthened with Circular Steel Jackets. Ph.D. Thesis.

[7] Zong Z.H., Deng J.D., Li Y., Liu A. (2013). Anti-seismic properties of damaged concrete bridge piers with hollow box-section strengthened with adhering steel plates I: Bi-directional quasi-static test. J. Southeast Univ. (Nat. Sci. Ed.).

[8] Chen Y., Feng H., Su C., Li X., Zhou G., Wang Z. (2025). Seismic behavior of earthquake-damaged hollow RC piers repaired with CFRP grids and ECC. Constr. Build. Mater..

[9] Huang H., Guo M., Zhang W., Huang M. (2022). Seismic Behavior of Strengthened RC Columns under Combined Loadings. J. Bridg. Eng..

[10] Benzaamia A., Ghrici M., Rebouh R., Pilakoutas K., Asteris P.G. (2024). Predicting the compressive strength of CFRP-confined concrete using deep learning. Eng. Struct..

[11] Sayed Y.A., Ibrahim A.A., Tamrazyan A.G., Fahmy M.F. (2023). Machine-learning-based models versus design-oriented models for predicting the axial compressive load of FRP-confined rectangular RC columns. Eng. Struct..

[12] Narlitepe F., Kian N., Demir U., Demir C., Ilki A. (2025). A novel hybrid thin jacketing method for seismic retrofitting of substandard reinforced concrete columns. Eng. Struct..

[13] Zhang R. (2024). Application of CFRP reinforced recycled concrete in structural research. Concrete.

[14] Wang P., Ma W., Zhao D., Wang C., Lu Y. (2026). Axial compression behavior of steel fiber-reinforced ultra-high-strength concrete filled high-strength square steel tube stub columns. J. Constr. Steel Res..

[15] Deng N., Liu X., Zhao H., Deng Y. (2025). Interfacial adhesion and optimization of ultra-high-performance concrete-filled steel tube columns containing welded rebar rings. Structures.

[16] Ma D.-Y., Guo Y.-Q., Han L.-H., Hou C.-C. (2025). Experimental investigation on the interface of concrete-filled steel tubular (CFST) structures based on roughness and geometric deviation. Eng. Struct..

[17] Abbas M.A., Ran X., Musleh A.A., Li P. (2025). Synergistic effect of expansive agent and steel fiber on interfacial performance and axial compression behavior of UHPC-filled steel tube columns. Constr. Build. Mater..

[18] Qu X., Zhang Y., Rong X. (2025). Seismic behavior and bearing capacity prediction of assembled concrete-filled multicellular steel tube composite shear walls with different horizontal joints. J. Build. Eng..

[19] Le H.A., Ho V.H., Nguyen P.-C., Le T.P., Lam M.N.-T. (2024). Axial compressive behavior of circular RC stub columns jacketed by UHPC and UHPFRC. Case Stud. Constr. Mater..

[20] Wang S., Fu Y., Ban S., Duan Z., Su J. (2025). Genetic evolutionary deep learning for fire resistance analysis in fibre-reinforced polymers strengthened reinforced concrete beams. Eng. Fail. Anal..

[21] Wei Y., Li K., Fan J., Zhu J., Jing D. (2025). Compressive and seismic performance of RC columns strengthened with steel strand meshes and ECC jacket: Analysis-oriented model and numerical investigation. J. Build. Eng..

[22] Hu Z., Elchalakani M., Yehia S., Ran H., Sadakkathulla M.A., Guo X. (2024). Engineered cementitious composite (ECC) strengthening of reinforced concrete structures: A state-of-the-art review. J. Build. Engineering.

[23] Wu H.Z., Emons C.D. (2021). Fiber Reinforced Polymer (FRP) Composites for Strengthening Concrete Structures: Design, Construction and Applications.

[24] Zheng Z.Y. (2018). Experimental Research on Seismic Performance of Damaged RC Column with Different Reinforced Measures. Master’s Thesis.

[25] Cao Q., Lv X., Li X., Gao R., Ma Z.J. (2021). Effect of self-stressing on concrete-encased-steel filled circular CFRP tubes under axial compression. Structures.

[26] Hou C., Ma G., Hwang H.-J., Li S., Kang S.-M. (2024). Seismic retrofit of intact and damaged RC columns using prefabricated steel cage-reinforced UHPC jackets and NSM GFRP bars. Eng. Struct..

[27] Cheng Z., Pei X., Hou H., Han T., Liu L., Wang H., Han Y. (2019). Expansive Behavior in Circular Steel Tube Stub Columns of SCC Blended with CFB Bottom Ashes. J. Mater. Civ. Eng..

[28] Shen P., Lu J.-X., Zheng H., Lu L., Wang F., He Y., Hu S. (2020). Expansive ultra-high performance concrete for concrete-filled steel tube applications. Cem. Concr. Compos..

[29] Huang W., Fan Z., Shen P., Lu L., Zhou Z. (2020). Experimental and numerical study on the compressive behavior of micro-expansive ultra-high-performance concrete-filled steel tube columns. Constr. Build. Mater..

[30] Cao Q., Li X., Lin Z., Wu Z. (2019). Compression behavior of expansive concrete-encased-steel filled square CFRP tubes. Compos. Struct..

[31] Ministry of Housing and Urban-Rural Development of the People’s Republic of China (2011). Specification for Mix Proportion Design of Ordinary Concrete (JGJ 55-2011).

[32] Xu W., Yang R., Zhao H., Li H., Zuo W., Liu J. (2024). Shrinkage separation prediction of concrete-filled steel tube arch bridge: A coupling model concerning multiple factors. J. Build. Eng..

[33] Li Y.-F., Geng Y., Ma H.-C., Zhang H. (2025). Creep behavior of composite steel tubular columns filled with weathered steel slag aggregate concrete. J. Build. Eng..

[34] (2009). Standard for Test Methods of Long-Term Performance and Durability of Ordinary Concrete.

[35] Wang K., Moon J., Du H., Xia X., Zhu D., Zhang P., Guo J. (2024). Early-age shrinkage and hydration of concrete incorporating a mutually reinforcing systems of super absorbent polymers and MgO expansive agent under low humidity conditions. Constr. Build. Mater..

[36] Shen D., Liu C., Wen C., Kang J., Li M., Jiang H. (2023). Restrained cracking failure behavior of concrete containing MgO compound expansive agent under adiabatic condition at early age. Cem. Concr. Compos..

[37] Wang D., Zhang G., Chen H., Hu Y., Qi D., Chen H. (2025). Investigation on mechanical properties of expansive lightweight aggregate ultra-high-performance concrete-filled steel tube short columns: Axial compression and interfacial bonding performance. Case Stud. Constr. Mater..

[38] Chen C., Chen R. (2022). Application of magnesium oxide expansive agent in hydraulic concrete in China. Mag. Concr. Res..

[39] Dhahir M.K., Marx S. (2023). Development of expansive concrete for chemical prestressing applications. Case Stud. Constr. Mater..

[40] Dassault Systèmes (2016). ABAQUS Theory Manual (Version 6.16).

[41] Ezoddin A., Azandariani M.G., Khatami S.M.H. (2025). Experimental and theoretical investigation on axial compressive bearing capacity of timber- and concrete-filled square steel tubes. Constr. Build. Mater..

[42] Han L.H. (2023). Principles of Concrete-Filled Steel Tubular Structures.

[43] Chen Z., Zhang K., Nong Y., Cai X., Tang J., Wang Y. (2025). Axial compression bearing capacity of concrete-filled circular aluminum tubular short columns: Calculation model and reliability analysis. Structures.

[44] Luo B., Wu M., Zhang X., Zhang P., Xiang P., Deng X., Huo J., Chen J., Xu S. (2024). Axial compressive bearing capacity of high-strength concrete-filled Q690 square steel tubular stub column. Constr. Build. Mater..

[45] Calderone A.J., Lehman D.E., Moehle J.P. (2000). Behavior of Reinforced Concrete Bridge Columns Having Varying Aspect Ratios and Varying Lengths of Confinement.

